# Fatty Acids: A Safe Tool for Improving Neurodevelopmental Alterations in Down Syndrome?

**DOI:** 10.3390/nu14142880

**Published:** 2022-07-13

**Authors:** Carmen Martínez-Cué, Renata Bartesaghi

**Affiliations:** 1 Departamento de Fisiología y Farmacología, Facultad de Medicina, Universidad de Cantabria, 39011 Santander, Spain; 2 Department of Biomedical and Neuromotor Sciences, University of Bologna, 40126 Bologna, Italy; renata.bartesaghi@unibo.it

**Keywords:** down syndrome, intellectual disability, neurodevelopment, cognition, therapy, fatty acids

## Abstract

The triplication of chromosome 21 causes Down syndrome (DS), a genetic disorder that is characterized by intellectual disability (ID). The causes of ID start in utero, leading to impairments in neurogenesis, and continue into infancy, leading to impairments in dendritogenesis, spinogenesis, and connectivity. These defects are associated with alterations in mitochondrial and metabolic functions and precocious aging, leading to the early development of Alzheimer’s disease. Intense efforts are currently underway, taking advantage of DS mouse models to discover pharmacotherapies for the neurodevelopmental and cognitive deficits of DS. Many treatments that proved effective in mouse models may raise safety concerns over human use, especially at early life stages. Accumulating evidence shows that fatty acids, which are nutrients present in normal diets, exert numerous positive effects on the brain. Here, we review (i) the knowledge obtained from animal models regarding the effects of fatty acids on the brain, by focusing on alterations that are particularly prominent in DS, and (ii) the progress recently made in a DS mouse model, suggesting that fatty acids may indeed represent a useful treatment for DS. This scenario should prompt the scientific community to further explore the potential benefit of fatty acids for people with DS.

## 1. Trisomy 21: Overview

Down syndrome (DS) is a relatively high-incidence chromosomal disorder (about 1 in 800–1000 live births) (see [1,2]) due to the triplication of chromosome 21. For this reason, it is also called trisomy 21 (T21). This gene burden causes a series of clinical manifestations, such as heart defects, hematologic, intestinal, and stomatognathic disturbances, and vision and hearing defects, which may not be present in all individuals with DS [3]. Intellectual disability (ID) present from infancy, however, is a constant hallmark of DS [4,5,6]. Although it exhibits a wide spectrum of severity, from very severe (IQ of 20–35) to mild (IQ of 50–70) [3], it prevents a completely autonomous life. Moreover, people with DS are particularly prone to developing Alzheimer’s disease (AD) starting from 35–40 years of age, which worsens an already compromised brain functioning because it leads to dementia [7]. ID is undoubtedly a major concern of the parents and guardians of children with DS and, additionally, it represents a social burden in view of its increase in during the last 10–20 years due to advances in medical care. Therefore, a pressing question is whether it is possible to ameliorate the damage to the cognitive abilities of people with DS, in addition to their general health, and to provide them with a greater degree of autonomy. In order to answer this question, it is necessary to know the causes of ID in DS.

In individuals with DS, the brain is smaller in comparison with the brain in the general population. This typical feature, which is particularly prominent in the cerebellum and hippocampus, is detectable in fetal life stages (see [8]). Early hypotrophy in the brain is not caused by a loss of neurons due to neurodegeneration but by impairment of the process of neuron generation (neurogenesis), which causes a −20 to −30% reduction in the number of neurons populating the brain (see [8]). Neurogenesis is reduced due to (i) a reduction in the proliferation rate of neural stem cells and progenitor cells (collectively, NPCs), and (ii) preferential differentiation of the newborn cells into astrocytes/oligodendrocytes rather than into neurons, i.e., a reduction in neurogenesis *sensu stricto* (see [9]). It must be noted that, although gliogenesis is not compromised in DS, astrocytes and oligodendrocytes exhibit various functional abnormalities that affect neuronal function [10,11,12]. This reduction in the number of neurons that populate the DS brain is worsened by the fact that their maturation is also compromised, which causes the generation of dendritic arbors scarcely ramified and endowed with dendritic spines that are reduced in number and have an abnormal shape [13,14,15,16,17,18]. Moreover, various neurotransmitter and receptor systems are altered in DS (see [19,20]), which, by building upon these ”gross” abnormalities, further impair brain-circuit functioning. At the cellular level, in addition to alterations to numerous pathways, mitochondrial, morphological, and functional alterations impair cell metabolism and cause oxidative stress [21,22]. These alterations may compromise neurogenesis due to an overall impairment in cell functioning [23,24] and may promote the precocious aging that characterizes individuals with DS [25]. Cell metabolic dysfunction, in turn, causes neuroinflammation, which further impairs neuronal functions [26]. Although the paucity of neurons and their aberrant maturation are most likely crucial determinants of ID in DS, all of the above-mentioned alterations promote ID. The term ID generically signifies an alteration in normal brain functioning. In the case of DS, ID includes various cognitive domains, among which the impairment of visual, spatial, and verbal memory, language, and executive behaviors stand out as particularly severe [27,28,29,30,31,32,33]. ID may be accompanied by psychiatric comorbidities, such as attention deficit hyperactivity disorder (ADHD), autism spectrum disorder (ASD), mood disorders, psychosis, and regression [34,35,36,37,38].

## 2. The Search for Therapies for DS and the Hypothesis: Can Fatty Acids Become a Therapeutic Strategy for DS?

The fact that an entire chromosome is triplicated makes it very difficult to establish which of the triplicated genes or sets of genes is responsible for the multifaceted alterations of the DS brain. However, accumulating evidence suggests that among the triplicated genes, *DYRK1A*, *APP*, *RCAN1*, *OLIG2*, and *DSCAM* are certainly major determinants of the neurodevelopmental alterations in DS (see [9]). The mechanisms regarding psychiatric comorbidities are less well understood, although alterations in GABA signaling and monoaminergic signaling are likely to play a prominent role (see [39]). Regarding AD development in DS, *APP* is thought to be the main candidate gene, although the mechanisms whereby increased *APP* gene dose causes AD in DS have only been partially clarified [40,41,42].

There are no treatments for ID in DS, no specific treatment for psychiatric comorbidities, and no treatment to prevent AD. During the last 20 years, great efforts have been undertaken to establish whether it is possible to improve ID in DS by taking advantage of murine models. Various mouse models of DS are now available (see [43,44]), among which the Ts65Dn mouse model has been extensively exploited because it exhibits numerous phenotypes that are typical of DS. These studies have shown that a variety of agents either targeting or not targeting triplicated genes can benefit cognition and may counteract age-related neurodegeneration, which encourages the search for treatments for DS (see [9,45,46,47,48,49,50]). Most of the treatments used in DS mouse models may raise safety concerns for human use, especially at early life stages. Natural substances have been traditionally used by humans to combat a myriad of illnesses and disturbances. Many natural substances are currently attracting attention as possible tools to ameliorate developmental brain disorders because they are able to change the expression of genes and the activity of pathways involved in brain development (see [51]). Accumulating evidence shows that fatty acids, which are nutrients present in a normal diet, exert many positive effects on the body and brain, affecting cardiovascular disease, diabetes, cancer, Alzheimer’s disease, dementia, depression, and visual and neurological development [52,53,54].

**Figure 1:**
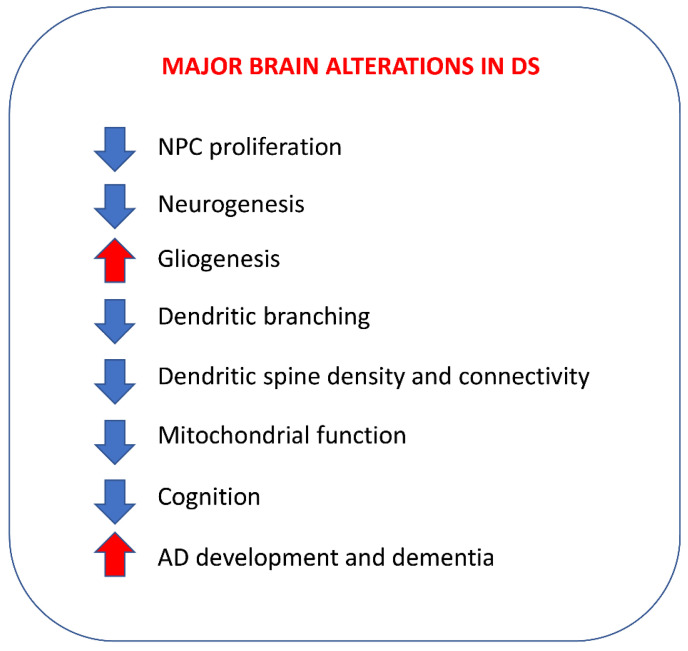
Summary of the major neurodevelopmental alterations in Down syndrome. See text for explanation and references. Abbreviations: AD, Alzheimer’s disease; NPC, neural progenitor cells. The upward pointing red arrows indicates an increase. The downward pointing blue arrows indicates a reduction.

The advantage of fatty acids is their generally safe profile, which is why they are useful during gestation and infancy. This is relevant for DS because the fetal period is the only time window during which cortical neurogenesis can be rescued. In this study, we review the knowledge obtained so far in various animal models regarding the impact of fatty acids on brain alterations that are particularly prominent in DS (Figure 1). This knowledge is fundamental for the design of future studies specifically devoted to establishing whether treatment with fatty acids may represent a useful tool for different DS-related phenotypes. Very few studies have explored the hypothesis that fatty acids may be a potential strategy for improvements in the neurodevelopmental and cognitive defects in DS. We also review recent achievements in relation to mouse models and individuals with DS, suggesting that fatty acids may indeed become a useful treatment for DS. These preliminary achievements should prompt researchers to further explore the potential benefits of fatty acids for people with DS. 

## 3. Overview of Fatty Acids: Chemistry and Nomenclature

**Figure 2:**
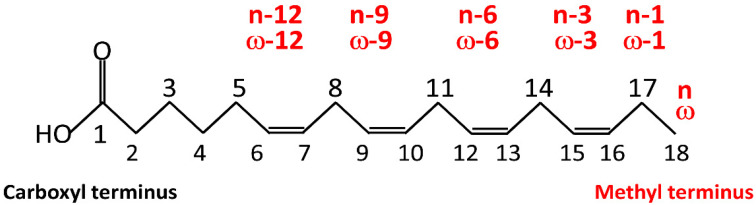
Naming of unsaturated fatty acids based on the position of the double bonds. According to one classification (IUPAC classification), this position refers to the distance from the carboxyl terminus, C1 (C-x). Alternatively, numbering refers to the distance of the double bond from the C atom of the methyl group, which is designated as n or ω. In n minus x (ω minus x), a double bond of the fatty acid is located on the xth carbon–carbon bond counting from the terminal methyl carbon (designated as n or ω) toward the carboxyl terminus. An 18-carbon FA is reported here as an example. The representation is idealized because, unlike saturated fatty acids, which have a straight-chain structure, PUFAs are typically bent.

Below, we provide a short description of fatty acids, mainly based on the reviews by Petrovic and Arsic [55] and Hussain et al. [56]. “Fatty acids” is an umbrella term including various chemical compounds, such as triglycerides, phospholipids, and cholesterol. Triglycerides make up 90% of dietary lipids and provide 30–40% of daily energy intake in Western societies. Triglycerides are the esters of glycerol and free fatty acids. Most fatty acids in the body take the form of glycerol esters. Fatty acids (FAs) are long hydrocarbon chain molecules (C4 to C28 atoms). Most FAs have an unbranched chain with a carboxyl group (–COOH) at one end and a methyl group (–CH3) at the other end. The naming of FAs is based on three main criteria. The first is the length of the hydrocarbon chain, which divides FAs into short-, medium-, long-, and very-long-chain FAs (Table 1). The second is the degree of unsaturation, i.e., the number of double bonds. Based on the degree of unsaturation, FAs are classified as either saturated if they have no C-C double bond (saturated fatty acids; SFAs) or unsaturated if they have at least one C-C double bond. Unsaturated FAs, in turn, are classified as monounsaturated FAs (MUFAs) if they have one double bond and polyunsaturated FAs (PUFAs) if they have two or more double bonds. The third is the position of the double bonds. According to one classification, this position refers to the distance from the carboxyl terminus, C1 (C-x). Alternatively, numbering refers to the distance of the double bond from the C atom of the methyl group, which is designated as n or ω (omega). In n minus x (also ω-x), a double bond of the fatty acid is located on the xth carbon–carbon bond counting from the terminal methyl carbon (designated as n or ω) toward the carbonyl carbon (Figure 2; see Table 2 for examples of FAs).

**Figure 3:**
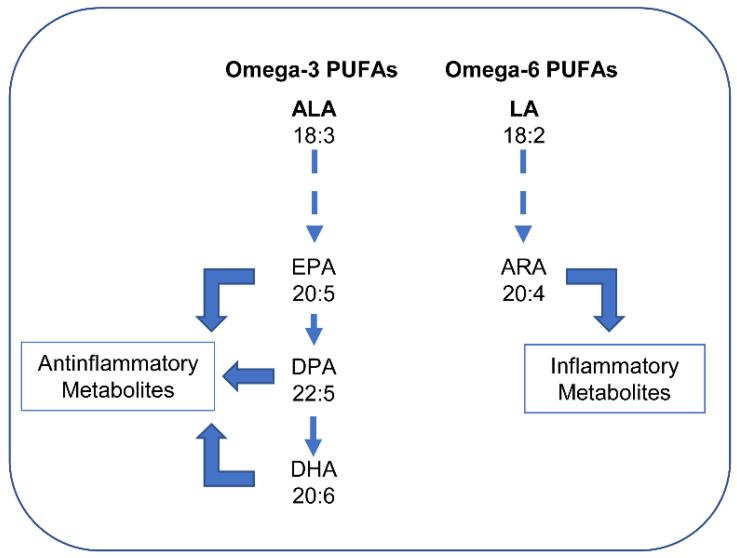
Synthesis of *n*-3 and *n*-6 PUFAs. ALA produces EPA, DPA, and DHA. LA produces ARA. *n*-3 PUFA-derived lipid metabolites largely inhibit inflammation. These mediators include resolvins and protectins. By contrast, ARA produces inflammatory metabolites (eicosanoids), which tend to promote inflammation. They include prostaglandins, leukotrienes, and thromboxanes (see [56,58]). ARA, however, also produces lipoxins that promote the resolution of inflammation (see [59]). Abbreviations: ALA, α-linolenic acid; ARA, arachidonic acid; DPA, docosapentaenoic acid; DHA, docosahexaenoic acid; EPA, eicosapentaenoic acid; LA, linoleic acid.

Unlike unsaturated FAs, SFAs are solid at room temperature. Thus, humans have FA desaturases that introduce a double bond. In addition, elongases increase the lengths of FAs. The most important PUFAs for humans are derived from two parent acids, linoleic acid (LA, 18:2 *n*-6) and α-linolenic acid (ALA, 18:3 *n*-3). Humans can synthesize SFAs and MUFAs, but they are not able to synthesize LA and ALA [57]. Both must be consumed with the diet and, therefore, they are termed “essential” fatty acids. Through the actions of desaturases and elongases, LA produces principally arachidonic acid (ARA, 20:4 *n*-6), whereas ALA produces mainly eicosapentaenoic acid (EPA, 20:5 *n*-3) and, subsequently, docosahexaenoic acid (DHA, 22:6 *n*-3). Lipid mediators derived from *n*-6 and *n*-3 PUFA are metabolically distinct and often have opposing physiological and pathological functions. While EPA, DPA, and DHA produce anti-inflammatory metabolites (e.g., resolvins), ARA produces pro-inflammatory metabolites (Figure 3). 

Thus, while *n*-3 PUFA-derived lipid mediators largely inhibit inflammation, *n*-6 PUFA-derived eicosanoids tend to promote inflammation (see [58,60]). However, it must be noted that not all metabolites of ARA are inflammatory. In particular, lipoxin A4, which derives from the metabolism of ARA, powerfully inhibits the synthesis of pro-inflammatory cytokines while increasing anti-inflammatory cytokines (see [59]). The overall effects of PUFAs are dependent on the *n*-3 and *n*-6 ratio. A ratio of 1:4 is recommended for health benefits to be obtained, although the ratio is about 1:10 in modern diets. Since the metabolites derived from *n*-6 and *n*-3 PUFAs have opposing physiological and pathological activities, the deleterious consequences associated with the consumption of *n*-6 PUFA-rich diets are thought to reflect the excessive production of *n*-6 PUFA-derived eicosanoids, while the beneficial effects associated with the consumption of *n*-3 PUFA-rich diets reflect the large production and activities of *n*-3 PUFA-derived mediators. 

Gut microbiota comprise trillions of microorganisms, including bacteria, archaea, and eukaryotes [61]. Gut microbiota can alter *n*-3 PUFA metabolism to generate increased levels of long-chain PUFA metabolites, such as EPA and DHA; furthermore, they can produce conjugated fatty-acid derivatives of PUFAs, such as conjugated linoleic acid (CLA) and conjugated α-linolenic acid, and they can increase the production of short-chain FAs (see [62,63]). Short-chain FAs are produced by microbiota through fermentation of nondigestible carbohydrates, such as dietary fibers. Among short-chain FAs, butyrate has multiple beneficial effects because it reduces mucosal inflammation, enhances FA oxidation, lowers total cholesterol, and prevents metabolic diseases [64,65]. 

In addition to inducing local changes in the gut epithelium, dietary metabolites derived from gut microbiota can affect multiple metabolic and physiological processes through epigenetic changes of gene expression (see [62]). Importantly, gut metabolites can influence the epigenetic machinery of brain cells, leading to changes in neuronal expressions that may cause changes in host behavior (microbiota–gut–brain axis). Consequently, any alteration in microbiota composition can lead to various disorders, such as inflammatory diseases, psychiatric disorders, and cognitive alterations [66]. Different factors affect the microbiota composition [67] and, thus, the epigenetic mechanisms that regulate gene expression. One of the most important factors affecting microbiota composition and microbiota-dependent epigenetic effects is the diet and the amount and type of dietary fat, with a high-fat diet being implicated in reduced gut microbiota richness [68].

## 4. Overview of Fatty Acids in the Brain

### 4.1. Major FAs in the Brain

Dietary fatty acids are currently of great interest because they act as signaling molecules responsible for a range of effects in the developing and adult brain [52,53,54]. Western fetuses accumulate FAs in the order of LA > ARA > DHA at all stages of pregnancy, and the highest accretion rates are reached in the last 5 weeks of gestation [59]. During pregnancy, most of the infant LA, ARA, and DHA are located in adipose tissue (68, 44, and 50%, respectively). Substantial amounts of LA (17%) and ALA (40%) are also located in skeletal muscle, and substantial amounts of ALA (11%) and DHA (23%) are also located in the brain [59]. Maternal omega-3 DHA and omega-6 ARA accumulate rapidly within the cerebral cortex during the last trimester of pregnancy, and the balance between DHA and ARA appears to play a fundamental role during brain development [60,61], as well as throughout life [62].

Approximately 50–60% of the adult dry brain weight consists of lipids, mostly structural lipids in the form of phospholipids (see [63]). These include large amounts of long-chain polyunsaturated fatty acids (LCPUFAs), mainly ARA and DHA, and very small amounts of LA and ALA. [57]. The lipids in the brain are both structural components and regulators of cell physiology. As components of plasma membranes, lipids provide fluidity and a framework for receptors, enzymes, transporters, and ion channels, and they function as reservoirs of lipid mediators. The LCPUFAs in the brain are important building blocks of neuronal membranes. The lipid bilayer of neuronal membranes consists of phospholipids, with DHA, ARA, and EPA as their main components. In addition to having a structural role, LCPUFAs support and improve brain structure and functioning. As detailed below, LCPUFAs (mainly in a healthy balance of *n*-6/*n*-3) play an important role during brain development by modulating neurogenesis, the outgrowth of axons and dendrites, and cognition, as well as normal aging by decreasing neuroinflammation, thereby preventing cognitive deterioration.

### 4.2. Sources of Transport of FAs in Neuronal Cells

Intracellular FAs may derive from (i) the lipolysis of complex lipids, (ii) de novo lipogenesis, or (iii) extracellular sources (see [69]). In the process of lipolysis, triglycerides are hydrolyzed by various lipases, producing FAs. In the process of de novo lipogenesis, fatty acid synthase (FASN) elicits the formation of palmitic acid (PA) from acetyl-CoA and, subsequently, malonyl-CoA. PA can then be expanded by elongases to produce stearic acid and/or desaturated to produce MUFAs, such as oleic acid (OA) (see [56,69]). Extracellular FAs are bound to fatty-acid-binding proteins (FABP), albumin, or in triglyceride form within apolipoproteins (Apo). Extracellular fatty acids can enter the cell by passive diffusion (short-chain FAs) or via transporters, such as FAT/CD36 or fatty-acid-transport protein (FATP) (see [69]). 

Since they have low solubility, FAs diffuse through the cytosol bound to proteins. Fatty-acid-binding proteins (FABPs) are a family of proteins that reversibly bind FAs and shuttle them to different intracellular compartments, such as mitochondria, peroxisome, endoplasmic reticulum, and the nucleus (see [70,71]). FABPs are expressed in NPCs in vivo and in vitro [72]. Fabp7 is expressed in embryonic and hippocampal NPCs and is necessary for the maintenance of NPCs [73]. Fabp7 is also required for the differentiation of neuronal and glial cells [72]. 

### 4.3. Functions of FAs in Neuronal Cells

#### 4.3.1. Components of Cell Membranes 

FAs can serve as components of neuronal membranes, into which they are integrated in the form of phospholipids or glycolipids. The types of FA (MUFAs or PUFAs) in the membranes contribute to their physicochemical properties, such as fluidity, which in turn may affect the functions of proteins embedded in the membranes, such as receptors or transporters.

#### 4.3.2. Production of Energy

FAs are stored as triglycerides in lipid droplets within cells. Following lipolysis, they enter the mitochondria for ATP production through β-oxidation.

#### 4.3.3. Signaling

FA signaling can be mediated by membrane and nuclear receptors. The membranes of peripheral and neuronal cells contain G-protein-coupled free-fatty-acid receptors (FFARs) (see [74]). Among the FFARs, there are two forms with prominent levels of expression in neural tissues: FFAR1 (also known as GPR40) and FFAR4 (GPR120 or O3FAR) [71]. Although the signaling pathways of FFAR1 and FFRAE4 in neural cells are not well understood, the effects of FFAR1 might be mediated by the phosphorylation of CREB (see [71]). In addition, FAs derived from membrane lipids can play a role as second messengers. For instance, diacylglycerol, which is formed by glycerol plus two FAs and is derived from the hydrolysis of membrane phosphatidylinositols operated by phospholipase C, can activate protein kinase C. The effects of FAs in the nucleus are mediated by the peroxisome-proliferator-activated receptors (PPARs). PPARs are ligand-activated nuclear-receptor proteins with a transcriptional function and represent the best-recognized nuclear sensor system for fatty acids [75,76]. There are three classes of PPAR: PPARα, PPARβ/δ, and PPARγ [76]. All three PPARs are able to bind FAs, with a general preference for LCPUFAs [75]. PPARs form heterodimers with their obligate partners, the members of another subfamily of nuclear receptors, the retinoid X receptors (RXRs). Upon ligand binding, PPAR/RXR heterodimers recognize and bind to specific DNA sequences, called PPAR response elements (PPRE). The chain length and degree of unsaturation of FAs affect their affinity to PPARs [77], which helps explain differences in the actions exerted by different FAs in the brain. 

## 5. Fatty Acids and Neurogenesis

The intrinsic genetic programs that regulate prenatal and postnatal neurogenesis can be modulated by many factors, including hormones, neurotransmitters, growth factors, cytokines, drugs, physical activity, and the environment (see [78,79]). Importantly, recent evidence indicates that FAs are not merely key elements of cell-membrane molecules or sources of energy, but rather appear to play a significant role in brain development by regulating NPC proliferation, differentiation, and maturation (see [56,69,71,80]).

### 5.1. Short Outline of Neurogenesis in Humans and Rodents

The brain is formed by neurons and glia (astrocytes and oligodendrocytes). The term neurogenesis includes two major processes: (i) the active proliferation of neural stem cells; and (ii) the differentiation of their daughter cells into either neurons or glia. The production of neurons and glia takes place during gestation. Neurons are produced first, followed by the production of astrocytes and, subsequently, of oligodendrocytes (for references and details regarding neurogenesis and gliogenesis, see [81,82,83,84,85]). Neurons and glia are derived from NPCs that are located in the germinative epithelium lining the wall of the neural tube. This epithelium is formed, initially, by the ventricular zone (VZ) and, subsequently, by the VZ plus the subventricular zone (SVZ). Before neurogenesis begins, NPCs divide symmetrically, forming two equal daughter cells, thereby expanding the pool of NPCs. At a certain time point, NPCs produce radial glial cells (RGC) that are NPCs themselves and divide asymmetrically, producing one RGC that remains in the VZ/SVZ and one neuron that migrates radially to the primordium of the future cortex. RGCs can also produce a population of cells called intermediate precursor cells (IPCs) or transit-amplifying cells (TAP), which, after a certain number of divisions, create neurons that migrate to the respective cortex. The formation of the cortex follows an inside-out pattern, according to which neurons migrating from the VZ/SVZ settle in increasingly superficial layers of the developing cortex. Cortical neurogenesis is completed by GW24–25 [84]. By contrast, cerebellar neurogenesis continues to the fifth postnatal month [86], while neurogenesis in the subgranular zone (SGZ) of the hippocampal dentate gyrus extends to the first postnatal year [82,87], and continues at a very slow rate throughout life [88,89,90,91]. Although cortical neurogenesis is completed before birth, the NPCs in the VZ/SVZ do not lose their proliferative capacity, but the neurons they produce are specifically destined for the olfactory bulb. In mice, cortical neurogenesis occurs between E11 and E17, hippocampal neurogenesis occurs between E10 and E18, and cerebellar granule cell neurogenesis starts at E13 and is completed at postnatal day 14 (see [46]). In rodents, the dentate gyrus produces most of its neurons (~80%) in the first two neonatal weeks and continues, slowly, to produce neurons throughout life. Similarly to humans, the postnatal VZ/SVZ of mice produce granule neurons destined for the olfactory bulb. 

The proliferation and differentiation of NPCs is controlled by genes that encode basic helix–loop–helix (bHLH) transcription factors [92,93]. Neuronal differentiation is promoted by activator-type bHLH factors, such as Mash1 and NeuroD, whereas repressor-type bHLH factors, such as Notch1, Hes1, Hes3, and Hes5, promote the proliferation of NPCs. In the absence of repressor-type bHLH factors, NPCs do not proliferate sufficiently, but prematurely differentiate into neurons and become depleted. Hes genes antagonize the activator-type bHLH genes that promote neuronal fate determination. The balance of activity among these factors is thought to determine cell fate [92,93]. 

### 5.2. Effects of Fatty Acids on Neurogenesis

#### 5.2.1. Overview 

Accumulating evidence shows that FAs play an important role in controlling neurogenesis in the two major forebrain neurogenic niches, the VZ/SVZ of the lateral ventricle and the SGZ of the hippocampal dentate gyrus [94]. 

Under normal conditions, the NPCs of the VZ/SVZ depend on fatty-acid oxidation for proliferation [95]. The NPCs of the SVZ do not require glucose to sustain aerobic respiration, unlike mature neurons and astrocytes. The NPCs within the adult SVZ oxidize fatty acids to produce energy, and this process is fundamental to supporting neurogenic activity. Indeed, the inhibition of fatty-acid oxidation decreases proliferation in cultured NPCs both in vitro and in the SVZ [95]. By contrast, the inhibition of fatty-acid oxidation does not decrease proliferation in the SGZ of the dentate gyrus. In this region, however, baseline and exercise-induced hippocampal neurogenesis require fatty-acid synthesis [96,97]. 

The relevance of FAs to the process of neurogenesis is highlighted by the fact that a doubling of membrane phospholipids is required in the S phase of mitosis for the creation of daughter cells [98]. Although the mechanisms whereby FAs affect proliferation are not well understood, emerging evidence (described below) shows that FAs are able to affect cell-cycle regulatory proteins and to change the expression of factors promoting NPC proliferation, e.g., Notch1 and Hes1, and differentiation, e.g., Mash1 and NeuroD [99,100,101,102].

Since the chemical structures of FAs may affect their function, and the cellular responses to FAs are characterized by distinct gene-expression profiles [103], we describe separately the effects of the different classes of FAs (SFAs, MUFAs, and PUFAs) on NPC neurogenesis.

#### 5.2.2. Effects of Saturated Fatty Acids

PA is the most common SFA, accounting for 20–30% of the total fatty acids in the human body. It has been considered detrimental to the health because it causes lipotoxicity [104], although its negative effects may be related to an imbalance of dietary PA/PUFAs [105]. PA appears to inhibit the proliferation of mouse-derived NPCs and favor astrogliogenesis, an effect associated with the activation of Stat3 (a transcription factor that promotes astrogliogenesis) [106]. In cultures of human embryonic stem cells, PA does not affect pluripotency or differentiation into neural progenitor cells, but reduces NPC proliferation [107]. Unlike in mouse-derived NPCs, however, PA increases the expression of genes needed for neuronal differentiation (e.g., *TBR2*, *TBR1*, and *NEUROD1*) and neurogenesis [107]. Adult mice that engage in voluntary exercise exhibit upregulation of hippocampal FA synthase, increased levels of PA (and stearic acid) in the hippocampus (but not the cortex or cerebellum), an increase in NPC proliferation in the SGZ of the dentate gyrus, and cognitive enhancement [96]. Taken together, experiments in vitro suggest that PA inhibits NPC proliferation [106,107] and favors gliogenesis in mouse-derived NPCs [106], but favors neurogenesis in human-derived NPCs [107]. It remains to be established whether these differences in cell fate are due to differences in the experimental protocols used in these studies or reflect differences between humans and rodents. Interestingly, the available in vivo evidence suggests that de novo synthesis of FAs, including PA, may be involved in the exercise-induced stimulation of neurogenesis in the adult dentate gyrus [96].

#### 5.2.3. Effects of Unsaturated Fatty Acids: MUFAs

Recent evidence shows that OA is the most abundant MUFA in human neural stem/progenitor cells and is necessary for their survival [108]. TLX (also known as NR2E1) is an orphan nuclear receptor that regulates the self-renewal and proliferation of NPCs [109]. OA was found to bind to TLX and to convert it from a transcriptional repressor to a transcriptional activator of cell-cycle and neurogenesis genes. This effect led to an increased proliferation rate of NPCs and their differentiation into neurons [108]. Nitro fatty acids (NO2-FAs) are naturally occurring compounds generated through the actions of reactive nitrogen species on cellular lipids, mainly PUFAs [110]. A recent study shows that nitro-oleic acid (NO2-OA) inhibits the expression of pluripotency markers (NANOG, SOX2, and OCT4) in murine embryonic stem cells and upregulates the expression of neuronal progenitor markers, such as Nestin [111], suggesting that OA, when nitrated, inhibits the proliferative potency of NPCs and enhances neurogenesis.

These studies show that OA increases the proliferation of NPCs and drives the progeny toward the neuronal lineage [108]. Although OA nitration inhibits stemness maintenance, nitrated OA retains the ability to favor the acquisition of a neuronal phenotype [111]. It must be observed that diets high in MUFAs decrease the number of proliferating NPCs in the hippocampus of adult male rats [112], suggesting that excessive doses of MUFAs may be deleterious for neurogenesis.

#### 5.2.4. Effects of Unsaturated Fatty Acids: PUFAs

LA was shown to favor the proliferation of mouse-derived NPCs [113], an effect that was accompanied by an increase in cell-cycle regulatory proteins (such as cyclin D1, cyclin E) and mediated via Ca2+, PLC/PKC, PI3K/Akt, and p44/42 MAPKs signaling pathways. In line with this study, LA was found to promote the maintenance of NPCs derived from rat embryos [114]. Treatment with safflower-seed oil, which contains a high concentration of LA (74%) (as well as 15% OA), was found to enhance the proliferation rate of mouse-derived cultured NPCs and, additionally, to favor neuronal differentiation [115]. LA alone, however, had no effects on proliferation or differentiation, suggesting the necessity of a synergic action of LA and OA (and/or other components of safflower seed oil). In line with this latter evidence, Hejr et al. found no effect of LA on either the proliferation or differentiation of mouse-derived embryonic NPCs [102]. The discrepancies between all these studies regarding the effect of LA on NPC proliferation may be explained by differences in the tissues used to derive NPCs and/or embryonic age of animals. Cis-9, trans-11-conjugated linoleic acid (CLA), is the primary isomer of LA naturally present in milk. In rat-derived embryonic NPCs, treatment with CLA was found to reduce the proliferation rate and to promote neuronal differentiation [101]. This indicates that LA and its major isomer may have opposite effects on proliferation, but that both may favor neurogenesis.

Hejr et al. found that ALA increased the proliferation rate of mouse-derived embryonic NPCs [102]. Treatment with LA had no effect, but various ratios of LA/ALA (1:3, 2:2, or 3:1) increased cell proliferation more than ALA alone, with the largest effect was at the ratio of 2:2 (LA 50: ALA 50), suggesting the relevance of a suitable ratio between these two PUFAs. Under differentiation conditions, the ALA increased the acquisition of an astrocytic and oligodendrocytic phenotype, but did not increase acquisition of a neuronal phenotype. ALA is the major component (90%) of Alyssum-homolocarpum-seed oil (AHSO). Mouse-derived embryonic NPCs treated with either AHSO or ALA were found to undergo an increase in proliferation rate and, under differentiation conditions, to preferentially acquire astrocyte and oligodendrocyte phenotypes [116]. Both these studies suggest that ALA has a pro-proliferative effect on NPCs and, under differentiation conditions, increases the acquisition of an astrocytic and oligodendrocytic phenotype. 

Kawakita et al. examined the effect of DHA on rat-derived embryonic NPCs [99,117]. They found that DHA reduced the number of proliferating cells, an effect that was associated with decreased levels of Hes1 (a transcription factor that maintains NSCs in an undifferentiated state), and increased the number of cells differentiated into neurons, an effect that was associated with increased levels of NeuroD, a neurogenic differentiation factor. The pro-neurogenic role of DHA was confirmed by another study [118] showing that in embryonic NPCs induced to differentiate, exposure to DHA increases the number of cells exhibiting a neuronal phenotype. The pro-neurogenic effect of DHA has also been found to be associated with the increased activation of WNT and CREB signaling [119]. Docosahexaenoylethanolamine (DEA, also called synaptamide) is a metabolite of DHA. Treatment with DEA was found to induce neuronal but not astrocytic differentiation of cultured rat NPCs, suggesting that endogenously produced synaptamide may promote neurogenesis [120]. 

Rats fed from postnatal day 2 until 31 days of age with a diet enriched with ARA, but not with DHA or ARA + DHA, were found to undergo an increase in the number of NPCs in the dentate gyrus, indicating that ARA, unlike DHA, exerts pro-proliferative effects [121]. Accordingly, in rats fed with ARA, but not DHA, there was an amelioration in the age-related decline in the number of hippocampal NPCs [122]. Under differentiation conditions, ARA was also found to promote the glial differentiation of NPCs [123]. Taken together, the studies on DHA and ARA suggest that DHA does not foster the maintenance of an NPC pool because it enhances neurogenesis. By contrast, ARA contributes to maintain the pool of NPC and does not favor neurogenesis, but favors gliogenesis.

ALA and DHA are omega-3 FAs, and LA and ARA are omega-6 FAs. The effects of different ratios of omega-3 and omega-6 FA may induce effects that are more complex than those elicited by ALA, DHA, LA, and ARA alone. In rats, a maternal diet deficient in ALA causes a reduction in brain phospholipid levels of DHA (which is derived from ALA) and an increase in the levels of ARA (which is derived from LA) in comparison with controls, with no change in proliferation in the SVZ and SGZ, but a reduction in neurogenesis [124]. An increase in dietary omega-3 relative to omega-6 fatty acids (2:1) favors neurogenesis and reduces apoptosis in the lobster brain [125]. In mice, a higher intake of omega-3 PUFAs with a lower ratio of *n*-6/*n*-3 PUFAs (between approx. 6:1 and 1:1) supplied to mothers during pregnancy favors NPC proliferation and counteracts apoptosis in the progeny [126]. Conversely, an increase in the ratio of *n*-6/*n*-3 due to a maternal diet deficient in omega-3 reduces neurogenesis in the offspring of rats [124,127].

#### 5.2.5. Conclusions

**Figure 4:**
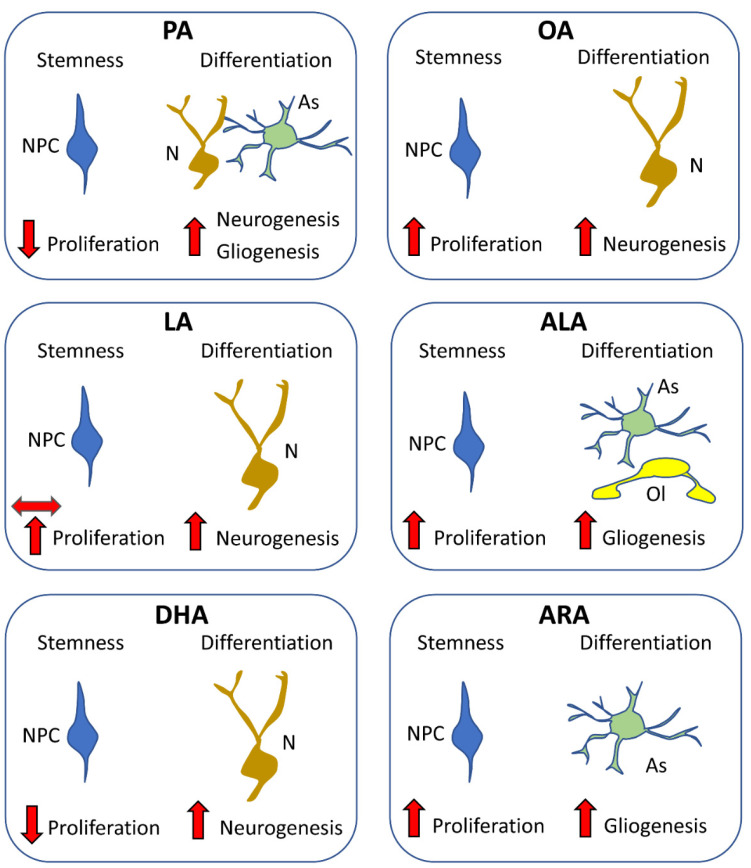
**Summary of the effects of fatty acids on neurogenesis**. Abbreviations: ALA, α-linolenic acid; ARA, arachidonic acid; As, astrocyte; DHA, docosahexaenoic acid; LA, linoleic acid; N, neuron; NPC, neural progenitor cell; OA, oleic acid; OL, oligodendrocyte; PA, palmitic acid. The double-headed horizontal arrow means no effect. The upward pointing arrow indicates an increase. The downward pointing arrow indicates a reduction. The double-headed horizontal arrow means no effect.

The data reported above show that different types of FA differentially affect the proliferation of NPCs and the differentiation of the progeny of NPCs into neurons or glia. The main effects of different FAs are summarized in Figure 4. These studies provide convincing evidence that fatty acids are useful for fundamental processes, such as energy metabolism and lipid membrane formation, but they could also serve as signaling molecules in neurogenic niches, where they regulate neurogenesis. NPCs appear to be sensitive to specific fatty acids. Suitable amounts and types of fatty acids may have a positive impact on NPC proliferation and neurogenesis, but excessive levels, improper ratios of different types of fatty acids, and disease-linked alterations to their metabolism may induce adverse effects.

## 6. Fatty Acids and Neuron Maturation

### 6.1. Overview of Neuron Maturation in Humans and Rodents

Once neurons are generated, they develop dendrites and an axon in order to establish appropriate synaptic connections. In humans, the development of the dendrites of cortical neurons starts at gestational week (GW) 13.5–GW15. An apical and basal dendritic tree is recognizable at GW17–GW25. Dendrites exhibit a growth spurt at GW26–GW32 [128,129]. At GW27, fibers from the thalamus invade the cortical plate [130]. Dendrites attain their maximum size between 4 months and 2 years of age [131]. Dendritic spines appear at GW25/26, and the process is completed by the end of postnatal months 5–6 [18,128,129,132].

In mice, an apical and basal dendritic arbor is already detectable in neocortical pyramidal neurons at postnatal day (PD) 2, but the apical arbor is less well developed than the basal arbor [133]. Similarly, in rats, the basal and apical dendritic arbor of CA1 pyramidal neurons has a different time course; the number of basal dendrites is already established at the age of 5 days, while the lateral and terminal branches of the apical arbor emerge later [134]. In rats, the apical dendritic branches of cortical pyramidal neurons increase in number up to PD15 [135]. Dendritic spines appear at approximately PD8 in both the hippocampus and the neocortex [133,135,136,137,138,139]. 

### 6.2. Effects of FA on Neuron Maturation

#### 6.2.1. Axon

Numerous studies show that OA synthesized by astrocytes fosters axon development in rat cultured neurons [140,141,142,143] and rat pups [144] and exerts this effect through the PPARα receptor [142]. Importantly, an oleate-rich diet restores the severe peripheral neuropathy induced in mice by a palmitate-rich diet [145]. Treatment with DHA fosters axon development in rat cultured neurons [146,147], and this effect is mediated by the activation of the Akt-mTOR-S6K pathway [147].

#### 6.2.2. Dendrites

In rat cultured neurons, OA increases neurite elongation, and this effect is prevented by inhibiting protein kinase C [148]. Ample evidence shows that DHA enhances neurite development in rat [117,146,149,150] and mouse [118] cultured neurons. Evidence in rat cultured neurons [151] and human iPSC-derived NPCs [119] shows that this effect is not mediated by nuclear retinoid X receptors (RXR) [151], but is associated with increased WNT and CREB signaling. [119]. Combined treatment with DHA and ARA also increases neurite development in cultures of human mesenchymal stem cells induced to differentiate into neurons [152]. ARA, EPA, and DHA exert robust effects on neurite development in cultures of primary sensory neurons from rat dorsal root ganglia, isolated at different ages; these effects are present from an early postnatal stage and persist into adulthood [153]. There is evidence that the neurite-outgrowth-promoting effect of NGF on PC12 cells is potentiated by DHA but reduced by ARA [154]. Another study, however, shows that DHA, EPA, and ARA (but not OA) enhance NGF-induced neurite growth in PC12 cells [155].

#### 6.2.3. Dendritic Spines and Synaptic Proteins

Growing evidence suggests that protein complexes that regulate spine plasticity depend on specific interactions with membrane lipids for proper function and accurate intracellular signaling (see [156]). In rat neuronal cultures, the local synthesis of OA increases the expression of synaptotagmin and postsynaptic density protein 95 (PDS-95), which are markers of the pre- and postsynaptic compartments, respectively [157], indicating that OA fosters connectivity. Ample evidence shows that treatment with DHA administered orally to rat dams during gestation and nursing [158], to adult gerbils [159], or supplemented to mice in neuronal cultures [118] increases the density of dendritic spines and the expression of pre- and postsynaptic proteins (Syntaxin-3, PSD-95, GluR-1, and Synapsin-1) and membrane phosphatides in the adult hippocampus [150,159,160]. By contrast, ARA has no effect on either dendritic spine density or membrane phosphatides [159,160], suggesting that the effect of DHA on spine formation may be related to enhancements in the synthesis of membrane phospholipids. 

#### 6.2.4. Conclusions

**Figure 5:**
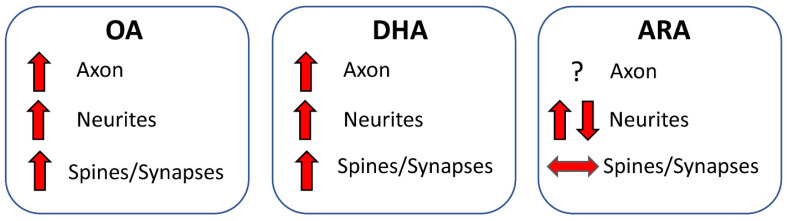
**Summary of the effects of fatty acids on neuron maturation.** Abbreviations: ARA, arachidonic acid; DHA, docosahexaenoic acid; OA, oleic acid. The upward pointing arrow indicates an increase. The downward pointing arrow indicates a reduction. The double-headed horizontal arrow means no effect.

Taken together, these studies suggest that OA favors both axon development and connectivity. DHA favors axon development, neuritic development, spinogenesis, and connectivity. No evidence is available for ARA regarding axon development. ARA may favor dendritic development, although results are not consistent across studies, and does not foster spinogenesis. The effects of different FAs are summarized in Figure 5. 

## 7. Fatty Acids and Mitochondrial Function

In humans, the highest concentration of LCPUFAs is found in the central nervous system. There is increasing evidence that, in addition to playing a structural role in membranes, LCPUFAs are crucial for energy production. LCPUFAs promote mitochondrial biogenesis [161,162] and modulate the expression of genes associated with ATP production and energy metabolism [163]. MUFAs may also have a positive effect on mitochondrial function because oleate prevents palmitate-induced mitochondrial dysfunction, insulin resistance, and inflammatory signaling in rat cortical neurons [164].

Brain regions with high energy consumption and high rates of oxidative metabolism have a high concentration of DHA and high levels of DHA in mitochondria [165,166]. In mice, DHA obtained from the diet is integrated into mitochondrial membranes [167], where it plays a crucial role in the mitochondrial oxidative phosphorylation system (OXPHOS), which is the biochemical pathway responsible for the production of energy in the form of ATP [165]. In addition, the mitochondrial respiratory chain, which is dependent on oxygen, generates reactive oxygen species (ROS). In fact, mitochondria are the main sources of ROS, since 90% of ROS is produced by the OXPHOS system as a byproduct of electron-transport-chain activity [166]. Impaired mitochondrial function is very commonly due to excessive ROS, affecting proteins of the OXPHOS system as well as the PUFAs of the mitochondrial membrane [168,169]. Moreover, alterations to the OXPHOS system produce carnitine deficiency, which impairs the mitochondrial pathway necessary for the synthesis of DHA-containing phospholipids [170].

During aging, the amount of mitochondrial cholesterol and phospholipid content decreases, membrane fluidity is reduced, and oxidative damage is enhanced [166,171]. In addition, mitochondrial respiratory capacity is decreased, and mitochondrial DNA (mtDNA) accumulates mutations due to enhanced ROS production [172,173]. Unfortunately, attempts to reduce mitochondrial decay by using antioxidants proved to have very limited efficacy [174]. Thus, it has been proposed that different compounds targeting other mechanisms underlying mitochondrial dysfunction could be useful. Among them, the supplementation of FAs to overcome their decay during aging could be a promising strategy. Indeed, FAs have been shown to reduce age-related mitochondrial alterations. Ochoa et al. compared the effects of the administration of MUFAs from virgin olive oil with those of the administration of omega-6 PUFAs from sunflower oil in aged mice [175]. While the MUFA diet led to oxidative-stress reduction and mtDNA deletions, the omega-6 PUFA diet increased lipid peroxidation in the mitochondria and caused an attenuation of mitochondrial membrane potential [175]. The administration of fish oil, which is rich in PUFAs, including the omega-3 DHA and EPA, attenuated mitochondrial dysfunction in aged mice [166]. Finally, supplementation with DHA had a protective effect in the mitochondria of a murine model of accelerated aging, the SAMP8 mouse [176], and protected against Aβ-mediated mitochondrial deficits in an AD animal model [177]. 

Oxidative-stress-related mitochondrial dysfunction may contribute to brain damage and neurodegeneration [178]. Several fatty acids have been demonstrated to induce neuroprotection. The oral administration of EPA inhibited irradiation-induced ROS production [179]. This neuroprotective effect seems to be mediated by the inhibition of JNK activation through the PPAR-α receptor, which suppresses the JNK-c-Jun pathways [180]. DHA protects from prenatal stress-induced cognitive dysfunction caused by alterations in mitochondrial function [181]. The beneficial effects of DHA are mediated by nuclear receptors, such as PPAR-α, HNF-4α, RXR, and LXRα, which induce changes in the expression of genes that encode transcription factors, fatty-acid-binding proteins, and inflammatory proteins [77,166,182]. 

Taken together, these data suggest that FAs, particularly LCPUFAs, play a physiological role in mitochondrial functioning and may improve mitochondrial dysfunction associated with aging and brain damage.

## 8. Fatty Acids and Cognition

A wide body of evidence relates FA to cognition. As mentioned in previous sections, LA and ALA are obtained from the diet. Maternal LCPUFAs are delivered to the fetal brain during the third trimester of pregnancy via the placenta and postnatally in breast milk. Deficits in maternal omega-3 FA, especially DHA, affect the metabolic and epigenetic factors of the fetus that deteriorate neurodevelopment and cognition [183,184]. When omega-3 deficiency is maintained across consecutive generations in rats, cognitive deficits are evident in the younger offspring [185]. There is a direct association between the percentage of fatty acids in maternal plasma and the development of cognitive function in neonates [186]. Moreover, maternal supplementation with DHA during pregnancy is associated with higher scores on tests of cognition in infants and preschool children, and a relationship between in utero DHA deprivation and several neurological birth defects has been proposed [187,188,189,190]. Deficits in DHA also produce cognitive deficits in humans during childhood and adulthood [183,191]. Among the cognitive domains affected by low levels of DHA are attention and working-memory deficits, reading difficulties, dyslexia, and dyspraxia. Children with attention deficit hyperactivity disorder (ADHD) and/or learning difficulties very commonly show low levels of omega-3 PUFAs in their blood [191]. Montgomery et al. evaluated the relationship between omega-3 PUFAs in blood and cognitive performance in children. They found that low omega-3 levels were strongly correlated with cognitive performance and proposed that supplementation with DHA or DHA-rich fish oil could reduce these deficits.

Animal studies have demonstrated the beneficial effects of supplementation with omega-3 PUFAs on mice or rats with cognitive impairments due to different causes. Supplementation with ALA, or its derivative, DHA, in the diet of pregnant female rodents enhances the cognition of their offspring [58,120,150,192,193,194,195,196,197]. The pro-cognitive effects of these diets were associated with the enhancement of neurogenesis, dendritogenesis, and synaptogenesis, suggesting that they were mediated by an improvement in brain development. Other processes, such as the restoration of the lipid composition of the membrane and reduced neuroinflammation, are also likely to be implicated in these cognitive improvements.

As mentioned in previous sections, the relative content of the different fatty acids is a critical factor determining the outcome of FA supplementation. Rats fed with oils that contain LA and ALA presented an improvement in spatial cognition in a Morris water maze, and the relative content of ALA appeared to be the critical variable [198]. High LA and low ALA supplementation for pregnant and lactating primates, mice, and rats leads to biochemical, neural, and cognitive impairments in their offspring [199], indicating that higher amounts of ALA in comparison with LA are necessary to induce cognitive improvements.

Given the positive effects of FA supplementation on animals’ cognitive abilities, it is expected that similar effects will be elicited in humans. Accordingly, many older adults consume omega-3 supplements to prevent cognitive decline. However, a systematic review and meta-analysis of randomized trials performed on adults, assessing the effect of higher vs. lower omega-3, omega-6, or total PUFA on cognition, did not confirm this presumption [200]. This meta-analysis suggested very low or no effect of omega-3 on cognitive impairment or global cognition. In addition, the effects of increasing the relative concentration of ALA, LA, or total PUFA were not clear. Thus, omega-3 PUFAs supplements do not seem to protect against cognitive decline.

## 9. Fatty Acids and Alzheimer’s Disease-Related Dementia

### 9.1. Altered Brain- Lipid Profile in AD

The most common type of dementia is caused by Alzheimer’s disease (AD). The etiopathology of this disease is multifactorial and includes enhanced oxidative stress and altered mitochondrial function, altered energy metabolism, neuroinflammation, reduced neurogenesis, loss of synapses, neurodegeneration, plaques composed of β-amyloid peptides, and neurofibrillary tangles of hyperphosphorylated tau protein. AD is also characterized by dysregulated FA composition and metabolism [69]. Studies have shown that long- and short-chain FAs play an important role in AD pathology, exerting both protective and pathogenic effects [201]. Because reduced levels of FA induce many of these pathological processes, it has been proposed that supplementation with medium-chain fatty acids (MCFAs) and omega-3 PUFAs may palliate the neuropathology and dementia found in mild cognitive impairment (MCI) and AD [202]. In fact, epidemiological and experimental research indicates that MCFAs and omega-3 PUFA supplementation can be beneficial in these diseases [56]. 

Elderly people consuming lower amounts of omega-3 FA have an elevated risk of AD, and several studies have implicated lower brain DHA in the pathogenesis of AD (see [190]). Moreover, post-mortem AD brains and plasma from patients with AD present lower levels of EPA and DHA [203,204,205]. In addition, an increased omega-3/omega-6 ratio negatively correlates with cognitive decline and the incidence of AD [206]. Furthermore, the level of PUFA oxidation products and the consequent oxidative damage is increased in AD brain tissue [207,208,209]. Interestingly, the main genetic risk factor for sporadic AD is a polymorphism of the ApoE gene [210], which encodes a lipid-carrying apolipoprotein in the brain. Alterations in lipid metabolism have also been found in animal models of AD, such as the 3 x Tg mouse [69]. These mice, as with humans with AD, present lipid droplets, which are intracellular stores of neutral lipids, such as triglycerides and cholesterol.

### 9.2. Supplementation with FA to Prevent or Reduce AD-Related Dementia

Several clinical trials have demonstrated that diets providing high levels of EPA and DHA are correlated with a reduced risk of developing AD [211,212,213]. However, controversial results have been obtained on the effectiveness of supplementation with FA at reducing dementia in MCI or AD. Some studies report that dietary supplementation with EPA and DHA improves cognitive performance in patients diagnosed with MCI or AD [214,215,216]. However, other studies failed to establish any benefit of omega-3 PUFA supplementation or cognitive improvements [217,218,219]. 

Despite the discrepancies, a wide body of literature suggests that FA supplementation might be beneficial in patients with MCI or in initial phases of AD in which dementia is relatively mild. These beneficial effects on the cognitive performances of patients have been attributed to: (i) an improvement in cerebral energy metabolism; (ii) a reduction in β-amyloid deposition, through a shift in the proportion of amyloidogenic and non-amyloidogenic APP processing; (iii) an increase in the clearance of Aβ42; (iv) a decrease in the aggregation of these peptides into amyloid plaques; (v) a reduction in oxidative stress and the enhancement of mitochondrial function and cerebral glucose metabolism; and (vi) a reduction in neuroinflammation (see [202]). The mechanistic effects of omega-3 PUFAs, especially DHA, on most of these mechanisms were reviewed in previous sections.

In conclusion, AD is characterized by altered homeostasis of brain lipids and changes in FA composition. Although studies conducted on animal models provided evidence that supplementation with omega 3-PUFAs could prevent or reduce cognitive decline in murine models of AD, the evidence in human trials is controversial (see [220] for a review). Nonetheless, most of the studies provide evidence for a potential benefit of these FAs in patients with MCI or in the early stages of AD. 

## 10. Fatty Acids and Down Syndrome

In view of the positive effect of FAs on a range of brain functions in healthy and diseased brains, it is surprising that scarce attention has been devoted to FAs in the context of DS. Below, we report what is known regarding FAs in the DS brain and discuss the results of pioneering studies that have explored the potential benefits of FAs for ID in DS.

### 10.1. Brain-Lipid-Profile Alterations in DS

Individuals with DS are characterized by impaired lipid metabolism [221] and alterations in the brain’s lipidomic profile, which are associated with a decrease in plasma-membrane fluidity, an important biological parameter affecting membrane biochemical and physiological properties [222]. The most frequent changes in the dyslipidemia of DS are low levels of high-density lipoprotein (HDL) and elevated levels of triglycerides (see [223]). The levels of carnitine, which transports long-chain FAs to mitochondria, are reduced in DS children [224]. This defect, in conjunction with impaired mitochondrial oxidative phosphorylation, may impair FA catabolism and, consequently, cause triglyceride imbalances. Individuals with DS exhibit reduced levels of some FABPs in the brain [225]. Since FABPs bind to PUFAs, stimulate cellular FA uptake, and enhance the intracellular diffusion of FAs, these reduced levels may also contribute to FA imbalance in DS. Whether alterations in the lipidic profile in DS are due to specific overexpressed genes on HSA21 remains to be established. *ABCG1*, a gene that encodes for proteins involved in the efflux of cholesterol from peripheral cells onto HDL [226], localizes to the long arm of chromosome 21 [227], suggesting that excess *ABCG1* may underlie some of the differences in lipid metabolism in DS. Individuals with DS exhibit a higher prevalence of diabetes, which is associated with dyslipidemia (increase in triglycerides and low HDL). Some HSA21 genes, such as *BACE2*, *RCAN1*, *DYRK1A*, and *S100B*, are thought to be related to diabetes phenotypes in DS (see [228,229]), suggesting that these HSA21 genes may also indirectly alter lipid metabolism.

The few available studies reviewed below show alterations in the plasma lipid profiles of women carrying fetuses with DS and in the brains of fetuses and adults with DS.

#### 10.1.1. Maternal Blood

The maternal blood of women carrying DS fetuses presents lower circulating high-density lipoprotein (HDL) in the first trimester of gestation and higher levels of low-density lipoproteins (LDL) during the second trimester of gestation [230]. In addition, during the first trimester, plasma-lipid extracts from DS mothers show decreased levels of 18:2 FA; during the second trimester, they present lower levels, of 20:4 and 22:6 FA [230].

#### 10.1.2. Fetuses with DS

Regarding MUFAs, reduced levels of OA have been detected in serine phosphoglycerides in the cortexes of fetuses with DS [231]. Albumin, which induces the synthesis and release of OA in astrocytes [232], is present at high levels in the brain during development [144,233,234]. DS individuals, however, present lower serum albumin concentrations [235,236], which might cause a lower synthesis of OA in this population. In cells from the T16 mouse model of DS, the uptake of OA is higher than in euploid cells; in the plasma membranes of euploid cells, an increase in phosphatidylcholine concentrations occurs in the presence of OA; and in trisomic cells, OA fails to increase phosphatidylcholine incorporation into the plasma membrane [234]. Regarding PUFAs, a study in DS and age-matched fetuses (during the fifth gestational month) quantified the PUFAs of ethanolamine and serine phosphoglycerides (EPG and SPG) [231]. The total percentage of PUFAs was similar in both groups. However, the ratio of omega-3 to omega-6 FAs was much higher in the DS fetuses. The levels of docosahexaenoyl, 22:6 (*n*-3) were enhanced, whereas the proportion of ARA 20:4 (*n*-6) and of its docosatetraenoic and docosapentaenoic homologues, 22:4 (*n*-6) and 22:5 (*n*-6), respectively, was significantly lower in the DS fetuses than in the controls. As DHA is more susceptible to peroxidation than ARA, an increased proportion of DHA in membranes could partially explain the excessive oxidative stress found in DS fetuses [231]. 

#### 10.1.3. Adults with DS

In adults with DS, the phospholipids in myelin and synaptosomes contain a reduced amount of MUFA and PUFA [237]. The total phospholipid content is reduced by approximately 20% in the cortex and cerebellum in adults with DS [238]. Small but significant differences were found in the proportion of several FAs between DS and age-matched controls (mean: 55.7 years) in the phospholipids of the frontal cortex [239]. In particular, choline phosphoglycerides in the DS frontal cortex exhibited an increase in OA (16:0) and 18:1 (*n*-7), a decrease in 18:0 and 18:1 (*n*-9), and small decreases in omega-6 PUFAs. Altogether, the ratio of total omega-3 to omega-6 PUFAs was increased in the DS cortices in comparison with the controls [239]. Adults with DS and AD undergo an increase in OA levels in the brain [239]; however, this is an adverse event that may promote AD development [240].

### 10.2. Effects of Fatty Acids in a DS Model

#### 10.2.1. Fetal Treatment

The evidence obtained in various cellular and animal models reviewed above (Section 5 and Section 6) shows that FAs exert beneficial effects on neurogenesis, neuron maturation, and synaptogenesis. As these processes are altered in DS, from the early stages of brain development, it can be hypothesized that the administration of FAs during critical developmental time windows (the prenatal and early postnatal periods) may improve these DS-linked alterations and, possibly, enhance cognition.

In a recent study, prenatal administration of OA or ALA to a murine model of DS, the Ts65Dn (TS) mouse, from embryonic day 10 (ED10) to postnatal day 2 (PD2), was found to rescue several trisomy-related neuromorphological alterations [241]. In particular, both treatments increased the brain weight, the volume of the granule cell layer (GCL), and the number of proliferating and mature granule cells in the hippocampus of PD2 TS mice, suggesting that treatment with either OA or ALA during embryonic brain development can foster prenatal brain neurogenesis.

In the same study, another cohort of mice was submitted to a washout drug-free period of 6 weeks after the discontinuation of the treatment. At this time, OA- and ALA-treated TS mice showed enhanced reference, working, and spatial memory in a hippocampal-dependent task (assessed with a Morris water maze) when compared to vehicle-treated TS mice. These results agree with the numerous reports of the pro-cognitive effects of the prenatal supplementation of OA, ALA, and its derivative, DHA, in different models of altered cognition (see Section 8). By contrast, adult TS animals did not differ from euploid mice in their GCL volume or in the number of proliferating cells. In the prenatal stages, brain development is regulated by the α-fetoprotein (AFP)/albumin ratio, which modulates the neurotrophic effects of oleic acid [242]. Thus, the balance between these two signals is crucial to induce neurogenesis during neurodevelopment. DS individuals present a reduction in the serum concentrations of AFP and albumin [236,243] that correlates with a lower concentration of OA in the brain [244]. In a study by Garcia-Cerro et al. [241], bovine serum albumin (BSA) was co-administered with OA (and ALA) to all animals. Thus, it is possible that during the administration of OA in the prenatal stages, adequate concentrations of this FA were reached, thereby correcting the impairment of hippocampal neurogenesis in the TS mice. However, after the discontinuation of treatment, the concentrations of both the OA and the ALA are likely to have returned to their previous deficit levels. This might explain why the beneficial effects of FA on neurogenesis were no longer present in TS mice after the discontinuation of treatment and suggests that the continuous administration of OA or ALA may be necessary to induce permanent effects on neurogenesis. 

Since the effects of OA and ALA on neurogenesis were not retained in the adult TS mice, the beneficial effects of prenatal treatment with OA and ALA on cognition cannot be attributed to permanent changes in neurogenesis. However, the TS mice did show an increase in the postsynaptic marker, PSD-95, in the hippocampus when compared to the vehicle-treated animals. PSD-95 is predominantly present in excitatory synapses [245,246]. OA promotes the expression of PSD-95 [157], which is implicated in long-term potentiation (LTP) [247]. TS mice are characterized by excessive over-inhibition due to an imbalance between GABAergic and glutamatergic transmission, which affects LTP and cognition [248]. Thus, an enhancement in the expression of PSD-95 induced by OA and ALA treatment could be implicated in improving the functioning of the brain circuits of TS mice prenatally treated with these FAs. 

Given that both OA and ALA are natural substances present in the human diet, and that they have no side effects, they could be safely administered prenatally, thereby fostering brain development and, possibly, improving cognitive abilities in DS infants.

#### 10.2.2. Early Postnatal Treatment

As previously mentioned, hippocampal neurogenesis continues after birth and, in rodents, it is very prominent in the early postnatal stages. Dendritic and synaptic development largely take place neonatally. Thus, the early postnatal period may provide a useful opportunity to enhance hippocampal neurogenesis and dendritic development in DS through treatment with FA. 

Based on this rationale, in a recent study, we analyzed the effect of the early postnatal administration (from PD3 to PD15) of OA or ALA on the hippocampal development and cognitive abilities of a TS mouse [249]. We found that neonatal treatment with OA or ALA did not exert any effects on the hippocampal NPC proliferation or the total size of the granule cell layer in the TS mice. These results are not in agreement with studies demonstrating that OA plays an essential role in postnatal neurogenesis from PD5 onwards [140,144]. However, consistent with the fact that OA also participates in neural differentiation, we found that early postnatal OA administration enhanced the percentage of the NPC that differentiated into neurons (BrdU+/NeuN+ cells) and the number of mature granule cells in TS mice. Thus, early postnatal OA treatment was sufficient to enhance differentiation but not proliferation in TS mice. Regarding ALA, neonatal treatment did not exert any effects on proliferation, differentiation, or survival, although it showed some benefit when prenatally administered [241]. Regarding cognition, while the early postnatal administration of OA improved reference, working, and spatial memory abilities when TS mice reached adulthood, treatment with ALA improved spatial memory only in the probe trial of the Morris water maze [249]. In view of the scarce (or absent) effects of treatment with OA or ALA on neurogenesis, the improvement in cognitive abilities found in treated TS mice’s cognitive abilities might be partially due to changes in synaptogenesis. Indeed, both OA and ALA increased the expression of PSD-95, which is implicated in LTP [247] and, thus, in learning processes. Moreover, OA and ALA might ameliorate other processes that are altered in the TS brain, such as oxidative stress, neuroinflammation, and altered energy metabolism, which might also contribute to the pro-cognitive effects of treatment. 

#### 10.2.3. Adult Treatment

As described in previous sections, brain development is affected not only by the total concentration of FA, but also by the ratio and relationships between them. Corn oil, which is extracted from the germs of corn, contains a high percentage of both LA and OA. LA is a precursor to the PUFAs of the omega-6 series, which produces ARA. Giacomini et al. [250] evaluated the ability of corn-oil administration for one month to young-adult (4-month-old) TS mice to improve neuromorphological and cognitive alterations. They demonstrated that adult treatment with corn oil restored hippocampal neurogenesis in TS mice. Moreover, the treatment enhanced dendritic length by increasing the number of branches of intermediate order and causing the *de novo* appearance of high-order branches. Importantly, the treatment enhanced hippocampal-dependent learning and memory (assessed with the Morris water maze and fear-conditioning tests), an effect that was very likely related to the restoration of neurogenesis and dendritogenesis. An evaluation of the separate effects of LA and OA in cultures of trisomic NPCs showed that both LA and OA increased their proliferation rate, although the effects of the LA were greater than those of the OA. The effects of the LA were mediated by the PPARβ/δ and PPARγ receptors, suggesting that these receptors might be involved in the effects of corn oil observed in vivo in TS mice. 

### 10.3. Effects of Fatty Acids on Individuals with DS

To the best of our knowledge, very few studies have been conducted to evaluate the effects of PUFAs on the DS population, and most of these studies evaluated the efficacy of PUFAs in association with other supplements, which makes it difficult to determine the relative contribution of PUFAs to the overall effects.

In one study, the biochemical responses of DS children after a nutritional regimen supplemented with amino acids, vitamins, and PUFAs were evaluated [251]. This nutritional supplementation regimen lasted 12 months and consisted of the oral administration of essential amino acids (acetylcarntine, citrulline, glutathione, histidine, alpha-ketoglutaric acid, methionine, ornithine, proline, serine, tryptophan, tyrosine, taurine, and hydrochloride betaine), vitamin B6, vitamin B12, vitamin C, vitamin E, and omega-3 and omega-6 PUFAs. This study was performed on 86 DS children and 80 healthy children divided into two groups, one ranging between 0 and 6 years of age and the other between 6 and 12 years of age. The study evaluated the plasma levels of amino acids, antioxidant enzyme activities, and ROS before and after supplementation. The authors reported that the supplementation they used had some benefits for the DS individuals, such as a recovery of the ratio of methionine to cysteine, the normalization of the levels of homocysteine (which was thought to have been produced by vitamin B6 and B12 supplementation), and a reduction in malondialdehyde, due to the antioxidant action of vitamins C and E. However, given the high amount of nutrients administered, this preliminary study provides little information regarding the contribution of PUFAs to the benefits found in this group of DS children.

Two other studies evaluated the safety and the effect of the green tea polyphenol epigallocatechin-3-gallate (EGCG) combined with fish oil omega-3 [252,253] on the mitochondrial dysfunctions that characterize the DS population. EGCG is a nutraceutical with antioxidant properties that also inhibits the activity of the protein product of the T21 gene DYRK1A. Although there are several reports regarding the efficacy of EGCG in animal models, and clinical trials have shown that administration of EGCG is safe and provides cognitive benefits in the DS population [254], treatment with EGCG has raised concerns because other groups have reported deleterious effects on bone or a lack of pro-cognitive effect of EGCG on the Ts65Dn model [255,256]. There is evidence that EGCG decreases saturated fatty acids (C17:0 and C22:0), increases unsaturated fatty acids (C18:1 and C18:2) in the serum, and decreases the liver-protein expression of SCD1 and FADS2 [257]. Thus, a lack of data on the individual effects of fish oil and EGCG prevents the determination of the synergistic/antagonistic effects of treatment with EGCG plus fish oil. Vacca et al. reported that the administration of EGCG and fish oil containing omega-3 FA EPA and DHA rescues mitochondrial dysfunction and is safe in a 10-year-old DS child [252]. As mentioned in previous sections, omega-3 FAs reverse mitochondrial dysfunction. In addition, in vitro studies have demonstrated the ability of EGCG to counteract oxidative stress, prevent OXPHOS deficits, and promote mitochondrial biogenesis [258]. Vacca et al. report that, after 3 months of supplementation, EPA and DHA enhanced EGCG bioavailability and its beneficial effects on mitochondrial function in a DS child without altering hepatic function and lipid profile. They also observed an improvement in auditory attention and verbal strategic tests [252]. In a pilot clinical trial, 14 DS children (1–8 years of age) received EGCG suspended in omega-3 FAs for 6 months, and 10 DS children and 6 healthy children who did not receive any substance were used as controls [253]. The authors reported that EGCG (>90%) plus omega-3 is safe in DS children and effective at reverting deficits in mitochondrial complex I and V activities. However, this treatment did not improve the developmental performance of DS children [253].

The scarcity of research and the fact that different agents were co-administered makes it difficult to draw conclusions regarding the effects of FAs in the DS brain. However, it seems reasonable to conclude that FAs might be involved in the improvement of mitochondrial function observed in subjects with DS and that treatment with FAs is free of adverse effects. 

### 10.4. Early Therapies with FA in DS: A Promising Strategy

**Figure 6:**
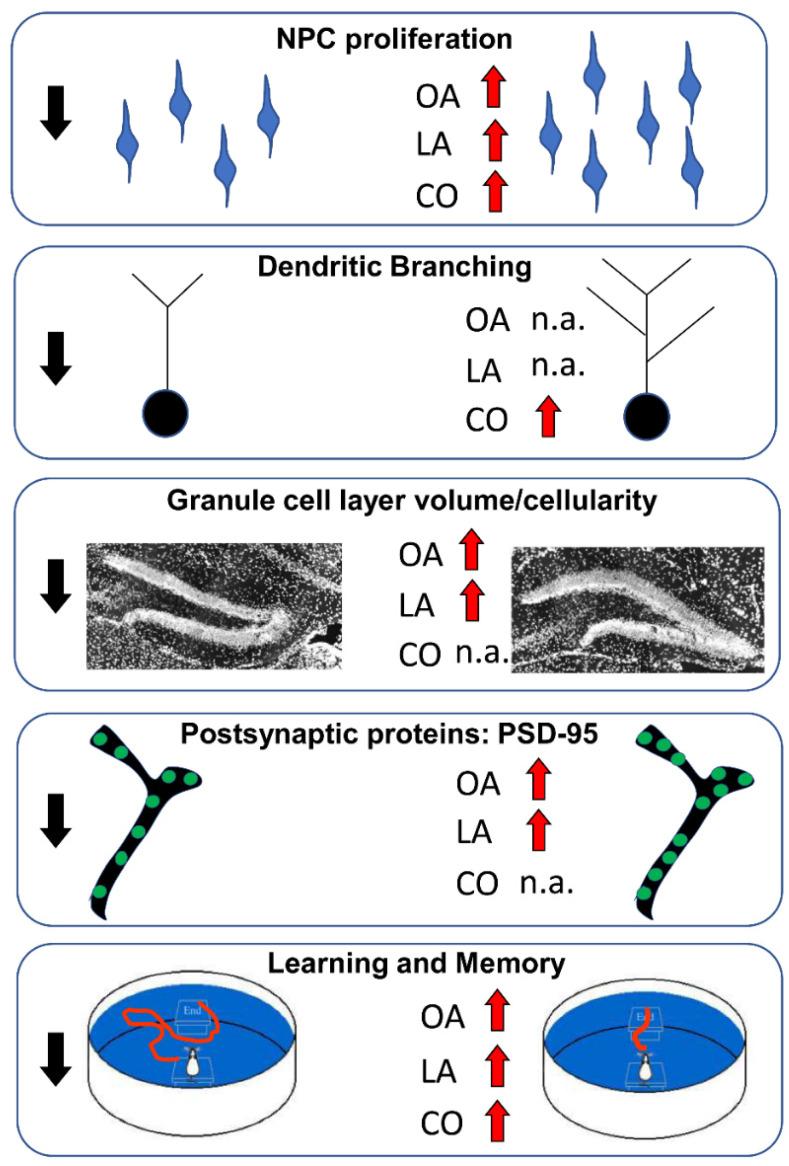
**Summary of the effects of fatty acids on brain development in the Ts65Dn model of DS.** The downward-pointing black arrows indicate trisomy-related impairment of the variables reported in each panel. The positive effects of treatment with fatty acids are indicated on the right by the upward-pointing arrows. See text for details. Abbreviations: CO, corn oil; LA, linolenic acid; n.a., not available; OA, oleic acid.

The results from a model of DS summarized above [241,249,250] and in Figure 6 provide the first demonstration that treatment with FAs may be a useful and safe strategy for the improvement of brain alterations and cognitive performance in DS. 

Taken together, the results of prenatal and neonatal treatment with OA and ALA [241,249] suggest that both exert positive effects on brain development, and some of these effects are retained in adulthood and translate into improved cognitive abilities. Prenatal treatment is much more effective than neonatal treatment, and treatment with OA is much more powerful than treatment with ALA. The superior effects of OA are possibly explained by a rescue of the intrinsic defects in OA levels/metabolism that characterize DS [144,231,232,234,235,236]. The data from adult Ts65Dn mice [250] suggest that a diet based on a combination of FAs may benefit the cognitive performance of DS individuals, even if administered during adult life stages. Given that these essential FAs are taken in the diet and that they are devoid of adverse effects, the provision of supplementation with these acids to the DS population could be a safe and promising strategy for improving DS cognitive alterations.

It remains to be established whether other types of FA may be more beneficial than those tested so far in the Ts65Dn model of DS. In relation to this, it is worthwhile to mention the results of a recent study showing that supplementation with fish oil reduces the levels of RCAN1 in normal mice pups [259]. *RCAN1* is one of the triplicated genes that is thought to be strongly involved in many DS brain phenotypes (see [9]). In line with the data on mice, the bioinformatic mining of human fish-oil studies also revealed reduced RCAN1 mRNA expression [259]. This study shows that FAs may target triplicated genes and supports the hypothesis that dietary supplements with FAs may represent a potential treatment for improvements in brain development in individuals with DS.

## 11. Concluding Remarks

The large body of evidence in animal models reviewed above shows that FAs are able to restore many of the brain defects that are found in DS, including neurogenesis, dendritic development, connectivity, mitochondrial function, neurodegeneration, and cognitive impairment. Thus, FAs may be rationally conceived as a potential tool for improvements in brain development and, possibly, the prevention/amelioration of AD in DS. 

Although the paucity of studies available for DS individuals prevents the determination of the correctness of this idea, the data obtained so far in a mouse model of DS clearly show that FAs restore or improve neurogenesis, dendritogenesis, connectivity, and cognitive performance, and that some of the effects of treatment leave a long-lasting trace on the brain. Therefore, we believe that these promising achievements should stimulate new research aimed at better delineating the effects of FAs. In view of the multifaceted effects of the large family of FAs on the brain, studies should examine the effects of different SFAs, MUFAs, PUFAs, and/or a combination of FAs, on the DS brain, in order to discover the optimal treatment.

New medical treatments should take into account two key prerequisites: efficacy and safety. In the case of DS, it must be remembered that neurogenesis failure is a key determinant of ID in DS and that cortical neurogenesis is completed before birth. Thus, treatment aimed at correcting neurogenesis failure should be administered prenatally to expectant mothers. This makes the issue of safety even more important, because the safety of both mothers and children must be taken into account. In this regard, natural substances may really become an option of choice because, at proper doses, they offer safety. We hope that this review may stimulate the scientific community to explore in depth the potential of FAs for DS, thereby providing a rational basis for new treatments.

## Figures and Tables

**Table 1:** Classification of fatty acids based on the length of the hydrocarbon chain.

Short-chain fatty acids	<8 carbons
Medium-chain fatty acids	8–14 carbons
Long-chain fatty acid	>16 carbons
Very-long-chain fatty acids	>22 carbons

**Table 2:** Examples of FAs.

Common Name	Abbreviation	C:D *n*-x
**Saturated fatty acids (SFAs)**		
Butyric		04:00
Caproic		06:00
Caprylic		08:00
Capric		10:00
Lauric		12:00
Myristic		14:00
Palmitic	PA	16:00
Stearic		18:00
Arachidic		20:00
**Mono-unsaturated fatty acids (MUFAs)**		
Palmitoleic		16:01 *n*-7
Oleic	OA	18:01 *n*-9
Erucic		22:01 *n*-9
**Polyunsaturated fatty acids (PUFAs)**		
α-Linolenic *	ALA	18:3 *n*-3
Stearidonic	SDA	18:4 *n*-3
Eicosatetraenoic	ETE	20:4 *n*-3
Eicosapentaenoic	EPA	20:5 *n*-3
Docosapentaenoic	DPA	22:5 *n*-3
Docosahexaenoic	DHA	22:6 *n*-3
Linoleic *	LA	18:2 *n*-6
γ-Linolenic	GLA	18:3 *n*-6
Arachidonic	ARA	20:4 *n*-6

Note: Number of carbon atoms in the fatty acid (C), number of double bonds (D), and the position of the first double bond counting from the terminal methyl carbon (*n*-x). EPA, DPA, and DHA derive from ALA and ARA derives from LA, in addition to being introduced with various types of food. ALA and LA (labeled with an asterisk) are essential FAs that must be introduced with the diet.

## Data Availability

Not applicable.
